# Real-world evidence: Risdiplam in a patient with spinal muscular atrophy type I with a novel splicing mutation and one *SMN2* copy

**DOI:** 10.1093/hmg/ddae052

**Published:** 2024-03-23

**Authors:** Kai Ma, Kaihui Zhang, Defang Chen, Chuan Wang, Mohnad Abdalla, Haozheng Zhang, Rujin Tian, Yang Liu, Li Song, Xinyi Zhang, Fangfang Liu, Guohua Liu, Dong Wang

**Affiliations:** Pediatric Research Institute, Children’s Hospital Affiliated to Shandong University, Jingshi road NO. 23976, Jinan, SD 250022, PR China; Department of neurology, Children’s Hospital Affiliated to Shandong University, Jingshi road NO. 23976, Jinan, SD 250022, PR China; Pediatric Research Institute, Children’s Hospital Affiliated to Shandong University, Jingshi road NO. 23976, Jinan, SD 250022, PR China; The Office of operation management committee, Central Hospital Affiliated to Shandong First Medical University, Jiefang road NO. 105, Jinan, SD 250022, PR China; Science, Education and Foreign Affairs Section, Children’s Hospital Affiliated to Shandong University, Jingshi road NO. 23976, Jinan, SD 250022, PR China; Pediatric Research Institute, Children’s Hospital Affiliated to Shandong University, Jingshi road NO. 23976, Jinan, SD 250022, PR China; Pediatric Research Institute, Children’s Hospital Affiliated to Shandong University, Jingshi road NO. 23976, Jinan, SD 250022, PR China; Pediatric Research Institute, Children’s Hospital Affiliated to Shandong University, Jingshi road NO. 23976, Jinan, SD 250022, PR China; Pediatric Research Institute, Children’s Hospital Affiliated to Shandong University, Jingshi road NO. 23976, Jinan, SD 250022, PR China; Ophthalmology department, Children’s Hospital Affiliated to Shandong University, Jingshi road NO. 23976, Jinan, SD 250022, PR China; Pediatric Hematology and Oncology, Children’s Hospital Affiliated to Shandong University, Jingshi road NO. 23976, Jinan, SD 250022, PR China; Intensive Care Unit, The Second People’s Hospital of Shandong Province, Duanxing west road NO. 4, Jinan, SD 250022, PR China; Department of Ultrasound, Central Hospital Affiliated to Shandong First Medical University, Jiefang road NO. 105, Jinan, SD 250022, PR China; Ophthalmology department, Children’s Hospital Affiliated to Shandong University, Jingshi road NO. 23976, Jinan, SD 250022, PR China; Pediatric Research Institute, Children’s Hospital Affiliated to Shandong University, Jingshi road NO. 23976, Jinan, SD 250022, PR China

**Keywords:** risdiplam, SMA, SMN1, SMN2, spinal muscular atrophy

## Abstract

Spinal muscular atrophy (SMA), which results from the deletion or/and mutation in the *SMN1* gene, is an autosomal recessive neuromuscular disorder that leads to weakness and muscle atrophy. *SMN2* is a paralogous gene of *SMN1*. *SMN2* copy number affects the severity of SMA, but its role in patients treated with disease modifying therapies is unclear. The most appropriate individualized treatment for SMA has not yet been determined. Here, we reported a case of SMA type I with normal breathing and swallowing function. We genetically confirmed that this patient had a compound heterozygous variant: one deleted *SMN1* allele and a novel splice mutation c.628-3T>G in the retained allele, with one *SMN2* copy. Patient-derived sequencing of 4 SMN1 cDNA clones showed that this intronic single transversion mutation results in an alternative exon (e)5 3′ splice site, which leads to an additional 2 nucleotides (AG) at the 5′ end of e5, thereby explaining why the patient with only one copy of SMN2 had a mild clinical phenotype. Additionally, a minigene assay of wild type and mutant SMN1 in HEK293T cells also demonstrated that this transversion mutation induced e5 skipping. Considering treatment cost and goals of avoiding pain caused by injections and starting treatment as early as possible, risdiplam was prescribed for this patient. However, the patient showed remarkable clinical improvements after treatment with risdiplam for 7 months despite carrying only one copy of *SMN2*. This study is the first report on the treatment of risdiplam in a patient with one *SMN2* copy in a real-world setting. These findings expand the mutation spectrum of SMA and provide accurate genetic counseling information, as well as clarify the molecular mechanism of careful genotype–phenotype correlation of the patient.

## Introduction

Spinal muscular atrophy (SMA) is a group involving several subtypes of neuromuscular disorders characterized by the degeneration of α-motor neurons in the anterior horn of the spinal cord, leading to symmetrically progressive muscle weakness and atrophy [1, 2]. SMA is still the leading cause of infant mortality worldwide with an incidence of 1 in 6000–10 000 live births [3, 4]. More than 95% patients with SMA are caused by decreased levels of the survival motor neuron (SMN) protein resulting from the homozygous deletions of at least exon 7 of the *SMN1* gene [5–7]. The remaining (less than 5%) cases are caused by compound heterozygosity variants of *SMN1*, with one *SMN1* deletion and one *SMN1* intragenic mutation, providing additional support for *SMN1* as a causative gene of SMA. The intragenic mutations in *SMN1* associated with SMA include missense, nonsense, or frameshift mutations located within the exons of *SMN1* and splicing mutations located in the intron regions of *SMN1*, which can lead to abnormal splicing of *SMN1* pre-mRNAs [8]. *SMN2*, a homologue of *SMN1*, can produce about 10% functional SMN protein of that of *SMN1*, partially compensating for the deficiency of *SMN1* [9]; therefore, the *SMN2* copy number in patients with SMA is inversely correlated with disease severity [10–12].

According to age of symptom onset and the maximum motor milestone that can be reached, SMA can be divided into five subtypes (types 0–IV) [13–15]. Among them, SMA type 0 is the most severe form of SMA, with a prenatal or neonatal onset. These patients require assisted breathing from birth and usually survive no more than 6 months [15]. SMA type I (OMIM #253300), also known as Werdag-Hoffman disease, accounts for about 50%–70% of patients with SMA, and it is a severe form of SMA, with symptoms usually occurring before 6 months of age [16]. Patients with SMA type I are unable to achieve higher motor milestones without treatment, such as sitting without assistance, and they generally have shortened lifespans [17, 18]. The affected patients usually exhibit a decline in motor function after symptom onset, accompanied with a decline of functions in respiratory and swallowing, and feeding support or combined feeding and ventilatory support typically needs to be provided to these infants by 12 months of age [16, 17]. SMA type II (OMIM #253500) is characterized by onset usually before 18 months [19]. The affected patients can sit independently, rarely stand, and only rely on others for support, but they cannot walk independently [20]. Their lifespan is shortened, with approximately 50% of individuals surviving to the age of 40 years [21]. Patients with SMA type III (OMIM #253400) have symptom onset beyond 18 months of age. They are able to walk independently with difficulty (waddling gait) but may lose this ability over time. As the condition progresses, they may require mobility support but generally with a normal life expectancy [20, 21]. SMA type IV (OMIM #271150), characterized by adult onset, is the mildest form of SMA, and individuals with SMA type IV have a fairly benign course of disease [19].

Currently, the U.S. Food and Drug Administration and the European Commission have approved three disease-modifying therapies for SMA. Nusinersen (SPINRAZA^®^), delivered by intrathecal injection, is an antisense oligonucleotide targeting the *SMN2* gene and has been approved for the treatment of both adult and pediatric populations with SMA [22, 23]. Onasemnogene abeparvovec (ZOLGENSMA^®^), given by intravenous infusion, is a gene replacement therapy-based viral vector, which is approved for the treatment of patients with SMA under 2 years old (US), or patients with SMA type I or 1–3 *SMN2* copies (EU) [24, 25]. Risdiplam (EVRYSDI^®^), administered orally, is a systemically distributed splicing modifier of *SMN2*, which increases the levels of functional SMN by promoting the inclusion of exon 7. It is indicated for children with SMA aged 2 months and older (USA) or aged 2 months and older with a clinical diagnosis of SMA types I, II, or III or 1–4 copies of *SMN* (EU) [26, 27]. The most appropriate individualized treatment for SMA has not yet been determined, and the best initial approach remains unclear [28]. In addition, the role of the *SMN2* copy number in patients treated with disease modifying therapies (DMTs) remains unclear.

In this study, we report a relatively mild case of SMA type I with a compound heterozygous variant in the *SMN1* gene: one deleted *SMN1* allele and a novel splicing variant c.628-3T>G in the retained allele and only with one *SMN2* copy. Further mRNA sequencing in *SMN1* gene showed that an insertion of two bases (AG) between exons 4 and 5 predicting will lead to a premature stop codon (p.Pro210SerfsTer4) but still retain partial normal spliced mRNA with c.628-3T>G variant, thereby explaining why the patient with only one copy of *SMN2* had a mild clinical phenotype. For the patient’s family to accept the treatment cost and proceed with drug treatment as early as possible, risdiplam was used for the treatment of this patient. However, the patient showed remarkable clinical improvement after treatment with risdiplam for 6 months despite carrying only one copy of *SMN2*.

## Results

### Genetic analysis

**Figure 1:**
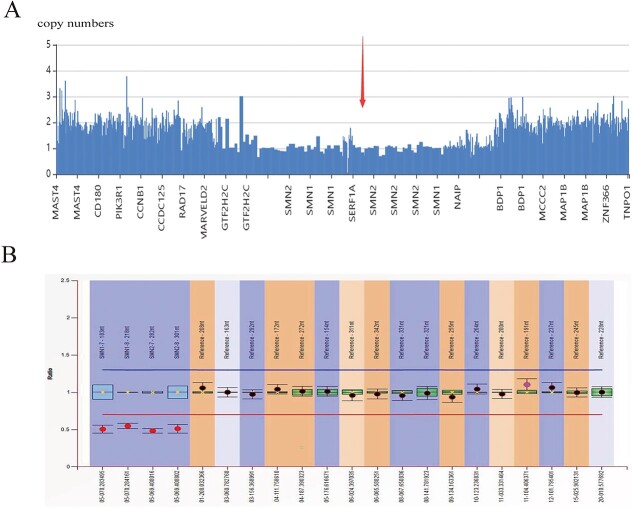
Genetic analysis of candidate *SMN1* and *SMN2* variants. (A) Result for CNV analysis of NGS indicating a loss of heterozygosity of *SMN1* and *SMN2* genes; (B) Results for MLPA, indicating the copy number of exons 7 and 8 of *SMN1/SMN2*.

**Figure 2:**
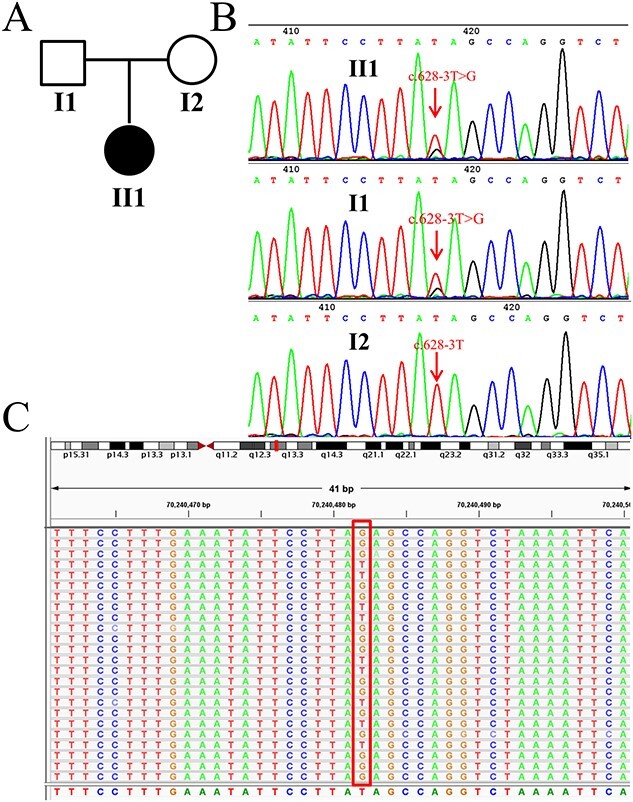
Identification of intragenic mutation in *SMN1*/*SMN2*. (A) Pedigree chart: Solid circle denotes the affected patient (II1). An open circle and square denotes the mother (I2) and father (I1), respectively. (B) Sanger sequencing showed that c.628-3T>G was the paternal variant. (C) Reanalysis of NGS data from the BAM file showing the mutation c.628-3T>G (only a portion of the data for all 384 reads is shown in the rectangular box).

Given the autosomal recessive genetic model and the clinical phenotype of neurological diseases, no highly suspected disease-associated subtle mutation was found by clinical exome sequencing. However, CNV analysis of NGS indicated the loss of heterozygosity of the *SMN1* and *SMN2* genes (Fig. 1A). MLPA was further employed to detect the copy numbers of *SMN1* and *SMN2*, and the results showed that both exons 7 and 8 of the *SMN1* and *SMN2* genes were absent (Fig. 1B). The results of NGS and MLPA analyses revealed that the patient carried only one copy of *SMN1* and *SMN2*. Given the result of loss of *SMN1* in one allele, we predicted that the patient might carry a subtle mutation in the other allele. Sanger sequencing identified a novel substitution of thymine by guanine at c.628-3 sit (c.628-3T>G) in intron 4 of the *SMN1/SMN2* gene in the patient (Fig. 2A and B). This mutation was predicted to change splicing of pre-mRNA in *SMN1/SMN2* and had a frequency of 0 in thousands of genomes, ESP6500, gnomAD, ExAC, and in-house databases. Given that *SMN1* and *SMN2* are two highly similar genes, both can be amplified as templates when using the common primers for *SMN*. With Sanger sequencing, the specific position of this novel mutation cannot be determined. Since there was initially no clear clinical diagnosis of SMA and *SMN1* and *SMN2* were highly homologous, the mutation c.628-3T>G did not come to our attention when analyzing the exome sequencing data. Thus, after the Sanger sequencing test, reanalysis of NGS data at this mutation site showed that the ratio of the number of mutated base G to wild T (a total of 384 reads) was approximately 1:1 (Fig. 2C). This result was additional proof for the loss of heterozygosity of the *SMN1* and *SMN2* genes.

### Bioinformatics analysis

**Figure 3:**
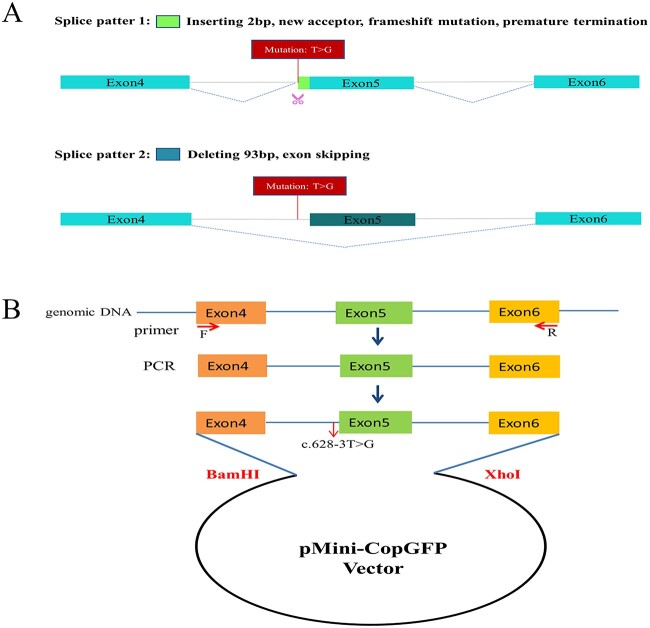
Schematic for the intronic mutation c.628-3T>G affecting splicing. (A) Online RNA splicer tool predicted two splice patterns: Pattern 1 with two base (AG) insertions between exons 4 and 5 and pattern 2 with exon 5 (96 bp) skipping. (B) Plasmid construction flow chart.

The novel variant c.628-3T>G in *SMN1/SMN2* was located at a splice receptor site, so in silico analysis including an online RNA Splicer tool and SpliceAI (a deep learning-based tool to identify splice variants) was employed to predict the possible effects on splicing. SpliceAI predicted a delta score of 0.87 for this variant. Two potential aberrant splice patterns were shown by the online RNA splicer tool: pattern 1 showed two bases (AG) were inserted at the 5′-end of the alternatively spliced exon 5 (Fig. 3A), which could lead to a premature termination codon (p.Pro210SerfsTer4); pattern 2 showed exon 5 (96 bp) skipping (Fig. 3A), suggesting that a new 3′ splice acceptor site (YAG|(Exon), Y = pyrimidine) formed in intron 4 with pattern 1.

### Splicing study of c.628-3T>G and identification of this mutation in the *SMN1* gene

**Figure 4:**
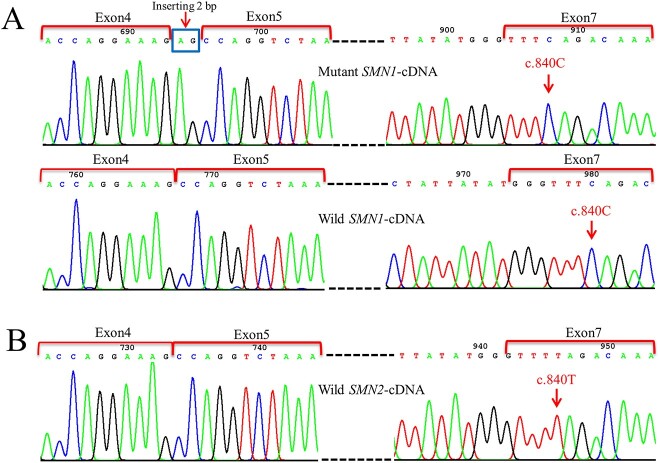
Splicing study of c.628-3T>G by sanger sequencing of SMN-cDNA clones. (A) Sanger sequencing of SMN-cDNA clones showing an abnormal splicing (2 bases AG was added at the 5′-end of the alternatively spliced exon 5 predicting will lead to a premature stop codon (p.Pro210SerfsTer4)) and a normal splicing in the *SMN1*-cDNA clones. (B) Sanger sequencing of SMN-cDNA clones, indicating normal splicing in all the *SMN2*-cDNA clones.

Given that *SMN1* and *SMN2* have only one copy confirmed by the above methods, *SMN1* and *SMN2* each can produce one form of mRNA. Therefore, c.628-3T>G could only have an abnormal splicing effect on one of the *SMN1* and *SMN2* genes. The splicing effect caused by c.628-3T>G in *SMN1* or *SMN2* was validated by Sanger sequencing of the SMN-cDNA plasmid described in the above method. A total of 14 randomly selected positive SMN-cDNA clones were used for Sanger sequencing validation, of which 10 were identified as *SMN2*-cDNA clones and the remaining 4 were *SMN1*-cDNA clones. In 4 *SMN1*-cDNA clones, 2 presented abnormal splicing (2 bases AG was added at the 5′-end of the alternatively spliced exon 5 predicting will lead to a premature stop codon (p.Pro210SerfsTer4)), but the other 2 showed normal splicing (Fig. 4A). All 10 *SMN2*-cDNA clones were identified as wild-type cDNA sequences without abnormal splicing (Fig. 4B). The retained normal-spliced *SMN1* gene can produce a portion of the SMN protein with normal function; thus, the patient’s phenotype did not become more severe. This aberrant splicing presented on *SMN1*-cDNA was consistent with splicing pattern 1 predicted by bioinformatics (Fig. 3A) and supported the patient’s clinical phenotype, proving that the mutation c.628-3T>G was in *SMN1* rather than in *SMN2*.

### Splicing study of *SMN1* c.628-3T>G by minigene assay

**Figure 5:**
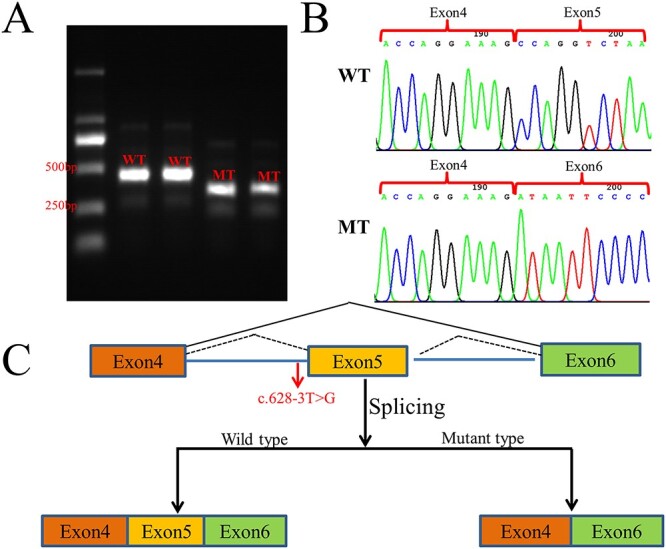
Splicing study of c.628-3T>G by minigene assay. (A) Agarose gel electrophoresis results of RT-PCR for plasmid expression: The wild-type fragment is estimated to be 451 bp, and the mutant type is estimated to be less than 400 bp. WT = wild-type; MT = mutant type. (B) Sanger sequencing of RT-PCR for plasmid expression. WT = wild-type; MT = mutant type. (C) Schematic of splicing for c.628-3T>G in the minigene assay.

To further characterize the altered splicing identified in vivo, we also conducted minigene analysis in the wild type and mutant type with *SMN1* c.628-3T>G (Fig. 3B). A single band from the wild type (expected 451 bp) was presented by agarose gel electrophoresis of the amplified products after RT-PCR, but the mutant type showed an unexpected band that predicted less than 400 bp (Fig. 5A). Sanger sequencing showed a normal splicing isoform from the wild type and an aberrant splicing with exon 5 deletion (96 bp) for the mutant type (Fig. 5B and C). This result was inconsistent with the splicing study from peripheral blood of the proband but consistent with the second prediction (Splice pattern 2) by the online RNA Splicer tool (Fig. 3A). However, this result did not support the clinical phenotype of the patient.

### Treatment

**Figure 6:**
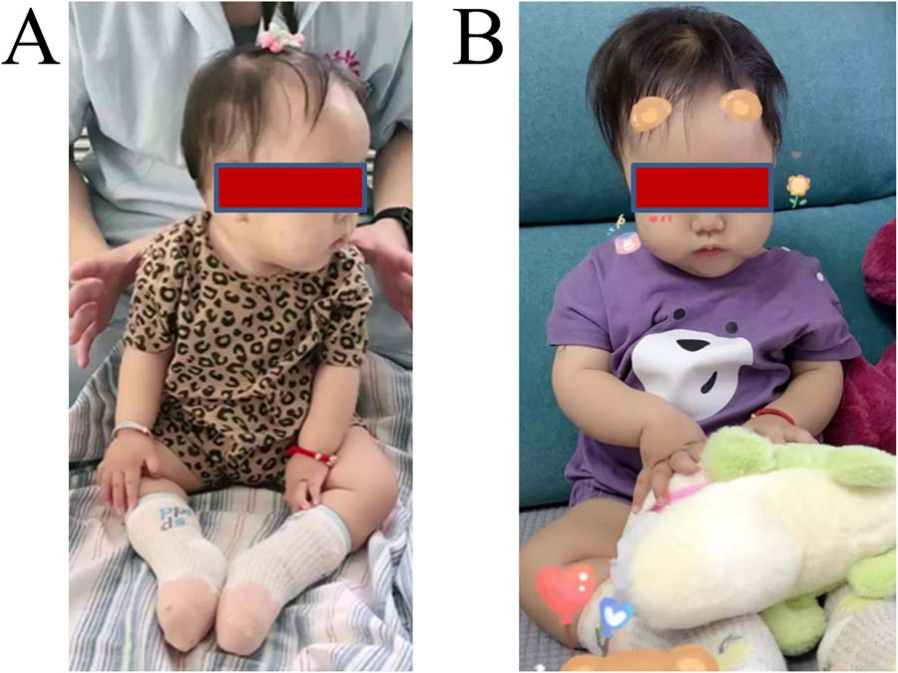
Demonstration of patient clinical improvement. (A) Display of the patient sitting for more than 5 s without assistance at 3 months post-risdiplam treatment. (B) Display of the patient sitting alone and playing with toys for 2 min at 3 months post-risdiplam treatment.

When the patient was 8 months old and had genetically acquired a definitive SMA type I diagnosis, she began to receive treatment with risdiplam. During the treatment, we evaluated the score of the patient’s Children’s Hospital of Philadelphia Infant Test of Neuromuscular Disorders (CHOP-INTEND), which is a reliable standard for assessing motor skills in patients with SMA and infantile neuromuscular diseases [29]. This measure contains 16 items of motor function, each rated from 0 to 4, including spontaneous movements of the upper and lower limbs, trunk, hip, and shoulder and head control. The total scores ranged from 0 to 64, with higher scores indicating better motor function. At 3 months post-risdiplam treatment, the patient showed moderate improvement in hypotonia, head control, and lower limb and hand prehensile strength. The patient maintained normal breathing and swallowing function and could sit unassisted for 5 s (Fig. 6A), with the CHOP-INTEND score increasing from 8 to 26. Such a remarkable improvement was beyond our expectations, as the patient had only one copy of *SMN2*. After 7 months of treatment, she could sit alone and play with toys for 2 min, with the CHOP-INTEND score increasing from 26 to 40 (Fig. 6B).

## Discussion

SMA is a leading monogenic cause of infant death worldwide. The estimated incidence of SMA ranges from 1:6000 to 1:1000, with a carrier frequency of 1:25 to 50 in most populations [3, 4, 30–32]. A total of more than 95% of patients with SMA (this proportion is lower in African black patients with SMA) present homozygous absence of at least exon 7 of the *SMN1* gene on chromosome 5q13, and the remaining patients (less than 5%) show compound heterozygous variants with an intragenic mutation on one allele and a deletion or gene conversion on the other [33–35]. *SMN2*, a nearly identical replicating gene to *SMN1*, has a variable copy number and can produce insufficient but essential levels of SMN protein. Given that an inverse relationship between copy numbers of the *SMN2* gene and severity of the SMA phenotype has been presented [8, 10, 36], assessment of the *SMN2* copy number in patients with SMA is essential to clearly elucidate genotype–phenotype correlations and predict the severity of the phenotype. To date, 147 variants have been recorded in the HGMD professional database, which included 111 intragenic mutations, with 56 missense/nonsense mutations, 14 splicing mutations, 40 frameshift mutations, and 1 regulatory mutation. Predicting the effects of intragenic mutations is usually not easy, which prevents a clear genotype-phenotype correlation, even though these mutations may be highly detrimental to the stability and function of SMN protein [37, 38]. Therefore, to establish a clear genotype-phenotype correlation for a patient with SMA and an intragenic mutation, two aspects of the effect of the intragenic mutation and *SMN2* copy number should be considered. Here, we identified a novel splicing variant c.628-3T>G in a patient with SMA type I and only one *SMN2* copy. We reviewed all 14 splicing mutations recorded in the HGMD professional database and found that the majority of these variants were located near exon 7 (12/14) [1, 33, 39–48], only one was in intron 3 [49], and the other was in intron 4 [50]. By contrast, the variant in our case was located in intron 4 (Table 1). All the 14 patients had one deleted *SMN1* allele (at least exon 7) in addition to splicing mutation allele. Half of all 14 cases did not have accurate splicing effect studies or *SMN2* copy number(s) that made it difficult to establish clear genotype–phenotype correlations. No. 1 case (Table 1), with one *SMN2* copy and a complete exon 7 skipping of *SMN1* transcripts caused by the splicing mutation of the single *SMN1* copy, presented a very severe phenotype of type 0 SMA. By contrast, our case, despite having only one *SMN2* copy, had a less severe clinical phenotype, which may be attributed to its low level of fully functional SMN protein from the *SMN1* transcripts. The two other cases (cases 4 and 14 in Table 1), with two *SMN2* copies, showed an unexpected mild SMA phenotype (SMA types III and IV). A similar explanation for this result was that the splicing mutations in cases 4 and 14 both induced a modest exclusion of exon 7 from the *SMN1* transcripts, and part of the normal functioning SMN protein from *SMN1* was retained. The above results showed that *SMN1* splicing mutations may contribute more significantly to the severity of SMA phenotype than *SMN2* copy numbers.

**Table 1:** Splicing mutations in *SMN1* that have been recorded in the current HGMD database.

**Case Number**	**Mutations (HGVS description)**	**Intron number**	**Effects**	**Phenotype**	***SMN2* copy number**	**Reference(s)**
1	c.*3+1G>C	Intron 7	Exon 7 skipping	Phenotype of type 0	1	41
2	c.*3+3A>T	Intron 7	Unknown	SMA type I	2	42
3	c.*3+4_*3+7delAGTC	Intron 7	Exon 7 skipping	SMA type II	Unknown	1;12
4	c.*3+6T>G	Intron 7	Exon 7 skipping with low levels of the full-length SMN	SMA type III	2	43
5	c.475-2A>T	Intron 3	Unknown	SMA type III	2	51
6	c.628-3T>G	Intron 4	Inserting 2 bp, resulting in frameshift mutation and premature termination with low levels of wild SMN	SMA type I	1	Our case
7	c.628-140A>G	Intron 4	Inserting 65 bp, resulting in frameshift mutation and premature termination	SMA type I	2	52
8	c.834+2T>G	Intron 6	Exon 6 skipping	SMA type I	Unknown	44
9	c.835-18_835-12delCCTTTAT	Intron 6	Exon 7 skipping	SMA type I	Unknown	1;12
10	c.835-1G>A	Intron 6	Exon 7 skipping	SMA type I	3	45
11	c.835-2A>G	Intron 6	Exon 7 skipping	SMA type I	2	46;48
12	c.835-2A>T	Intron 6	Unknown	SMA type I	Unknown	47
13	c.835-3C>A	Intron 6	Exon 7 skipping	SMA type I	2	48
14	c.835-3C>T	Intron 6	Exon 7 skipping but retains most of the full-length SMN	SMA type IV	2	49
15	c.835-5T>G	Intron 6	Unknown	SMA type I	2	50

**Figure 7:**
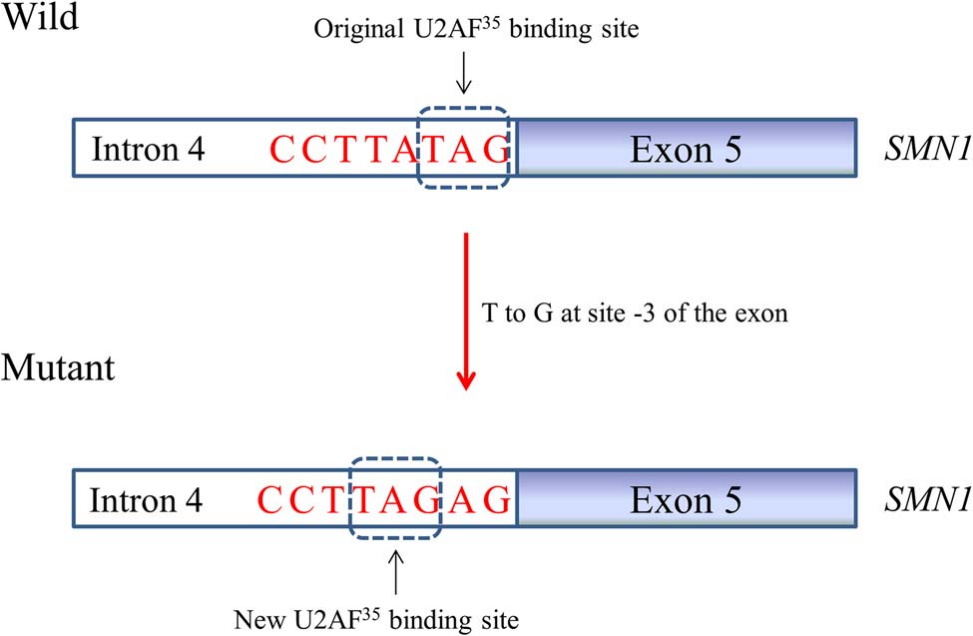
Schematic of the new splice acceptor site generation.

Interestingly, cases 13 and 14 both had two *SMN2* copies and one mutation at the same site 3 nucleotides upstream of exon 7 in *SMN1* (site c.835-3 of *SMN1*, case 13 with c.835-3C>A and case 14 with c.835-3C>T), but the clinical severity of the two varied greatly (case 13 with SMA type I and case 14 with SMA type IV); thus, splicing mutations caused by different base substitutions at the same site may have various effects on splicing. According to the consensus for splice acceptor sites [51], the three-nucleotide sequence YAG|(Exon) upstream of exon near the intron-exon junction (positions −3, −2, −1|(Exon) of the exon, Y = pyrimidine), as one of these sites, is recognized by U2AF^35^. Therefore, case 13 featured a severe clinical phenotype, probably because the mutation changed the pyrimidine of the nucleotide at position −3 of the exon from cytosine to adenine (C-to-A). Although the mutation in case 14 did not change the nature of the pyrimidine, the authors argued that c.835-3C>T probably induced the exclusion of exon 7 from *SMN1* transcripts by reinforcing a hnRNP A1 binding site that spanned the acceptor site of the exon [47], so it had minimal effects on splicing and the case had a mild clinical phenotype. Nevertheless, a mutation at position −3 of the exon from a pyrimidine to a purine leads to unstable binding of U2AF^35^ to this site that allows spanning the acceptor site of the exon, which may cause a harmful effect on the splicing of the gene’s pre-mRNA. Notably, the mutation c.628-3T>G in our case was also at site −3 of the exon; only the insertion of two bases (AG) and a small amount of normal splicing but no exon skipping were found in the in vivo study. One reliable explanation is that this mutation created a new splice acceptor site (YAG) that could be stably bound by U2AF^35^, but a small portion remained bound to the original site (Fig. 7). Although the minigene splicing assay is considered a reliable, effective and relatively simple tool to functionally analyze potential splicing [52], surprisingly, in vitro minigene assay with the mutation c.628-3T>G detected a complete exon skipping that was inconsistent with our in vivo study. Exons present in heterologous environments may not necessarily characterize the exact splicing profile of the endogenous gene [53]. This result suggested that the findings can be confirmed by using RNA from patients whenever possible.

In generall, the *SMN2* copy number has been used to predict disease severity, but the role of the *SMN2* copy number in patients treated with DMTs is unclear. Nowadays, three molecular therapies (onasemnogene abeparvovec, nusinersen, and risdiplam) with different therapeutic mechanisms and administration routes have been approved for the treatment of SMA. As a result of the lack of comparative data among these approved molecular therapies, the most appropriate individualized treatment for SMA has not yet been determined. Risdiplam (Evrysdi™), as an oral RNA splicing modifier targeting *SMN2*, has been approved for the treatment of patients with SMA or a clinical diagnosis of SMA type 1, 2 or 3 or 1–4 *SMN2* copies (EU) [26, 27]. However, we reviewed four current ongoing clinical trials assessing risdiplam: the FIREFISH (NCT02913482), the JEWELFISH (NCT03032172), the RAINBOWFISH (NCT03779334), and the SUNFISH (NCT02908685) trials [54–58]. We found that almost no patients with one *SMN2* copy were included in their study, or if they did, it would not provide a detailed reference for individualized treatment. Our study is the first report on the treatment of risdiplam in a patient with one *SMN2* copy in a real-world setting. On the basis of the functional skills reported by the patient’s parents and CHOP-INTEND assessment, the patient showed remarkable motor improvement. We suppose that this favorable clinical response was due to the fact that the patient in our study had higher concentrations of fully functional SMN protein than those who had complete deletion of the *SMN1* gene but only two copies of the *SMN2* gene. Therefore, a slight increase in the concentration of fully functional SMN protein after treatment with risdiplam could lead to good clinical improvement. However, a limitation of this study is that SMN protein concentrations were not measured before and after treatment with risdiplam. No laboratory abnormalities or major side effects were detected. Assessment of the long-term efficacy and any delayed onset side effects of risdiplam is ongoing by comprehensive follow-up data collection.

Overall, first, this study is the first report on the treatment of risdiplam in a patient with one *SMN2* copy in a real-world setting. Our findings expand the mutation spectrum of SMA, provide accurate genetic counseling information, and clarify the molecular mechanism of careful genotype–phenotype correlation of the patient. Second, our work highlights that the functional analysis of splicing variant is important to establish careful genotype–phenotype correlations and predict disease severity, particularly to evaluate their effects on RNA splicing. Meanwhile, our findings underscore the importance of using in vivo assays to accurately determine the effect on RNA splicing of patients with SMA, as in vitro assays may not accurately reflect splicing results in patients. Third, functional analysis of intragenic mutations in patients with SMA may provide some guidance for the selection of individualized treatment for patients. Finally, our study showed that risdiplam could be effective in patients with SMA type I who have only one copy of *SMN2* but with high levels of functional SMN protein from *SMN1*.

## Material and methods

### General information and clinical examination of the patient

The propositus, a female infant, was born uneventfully to non-consanguineous parents following a normal pregnancy and delivery without family history of known genetic neurological disorders. As reported by the patient’s parents, she presented a symptom of limb weakness when she was 3 months old. The parents took her to the local hospital, but no confirmed result was obtained. The patient visited the neurology department of our hospital when she was 5 months old. She presented with diffuse muscle hypotonia and had not yet achieved the expected head control and turn over motor milestones.

Neurological examination of the patient revealed decreased movement and hypomyotonia of limbs and trunk. The muscle strength of the upper limbs was grade III, whereas that of the lower limbs was grade II. The reflexes of bilateral biceps, triceps tendon, and ankle were not elicited. No signs of chest malformation or respiratory distress were present in the patient. She had normal swallowing function without fever disease. Electromyography showed extensive neurogenic damage, possibly damage of the spinal cord anterior horn. Laboratory examinations showed normal results of the muscle enzyme spectrum, including creatine kinase (CK), creatine kinase MB (CK-MB), aspartate transaminase (AST), and alanine aminotransferase (ALT). Considering the clinical manifestation, neurological examination, and electrophysiological results, our clinical diagnosis was congenital neurological disorder, with emphasis on SMA type 1.

### Next-generation sequencing (NGS) and variant calling

For the consideration of a genetic neuromuscular disease, NGS was applied to screen the genetic cause of the patient. Peripheral blood from the patient and her parents was collected in an EDTA vacutainer. The QIAamp DNA Blood Midi Kit (Qiagen, Shanghai, China) was used for genomic DNA extraction of blood samples. A NanoDrop 2000 ultraviolet spectrophotometer (Thermo Fisher, USA) was used to determine the extracted DNA. GenCap MedE006 capture kit (MyGenostics, Beijing, China) was applied for clinical exome sequencing to screen for variants in the probands with a HiSeq X ten sequencer (Illumina, United States). After sequencing, the raw data were exported and stored as a FASTQ format. Low quality sequences, such as Illumina sequencing adapters and low quality reads (< 80 bp), were filtered using cutadaptor software (http://code.google.com/p/cutadapt/). The clean reads were mapped to the UCSC human reference genome (version GRCh37/hg19) with the BWA parameter in Sentieon software (https://www.sentieon.com/). A BAM file was then generated. The driver parameter of Sentieon software was used to mark and remove the duplicated reads and correct the base. The quality of corrected bases can reflect a real mismatch probability with the reference genome. The driver parameter of sentieon software was used to locally reassemble the active region (that is, regions that are more different from the reference genome) and call the SNPs and InDels variants. The data were converted to VCF format. ANNOVAR software (http://annovar.openbioinformatics.org/en/latest/) was further used for annotation of variations. The database of normal human genomes was used to determine the frequency of mutations. Variant frequencies were determined in 1000 Genomes Project, Genome Aggregation Database (gnomAD), Exome Aggregation Consortium (ExAC), Exome Sequencing Project 6500 (ESP6500), and in-house databases to remove common variants (sub-allelic frequency > 5%). Potential disease-causing variants associated with the patient-standardized HPO phenotype were prioritized for this study. The pathogenicity of novel variants was predicted by SIFT, MutationTaster, PolyPhen-2, and GERP++. Segregation analysis of the suspicious variations was performed by Sanger sequencing. Whole genome copy number variation (CNV) analysis was conducted with the FASTQ format and BAM file based on the above methods. The CNV information was obtained with CNVkit software (https://cnvkit.readthedocs.io/en/stable/).

### Multiplex ligation-dependent probe amplification (MLPA) analysis

Blood samples were collected from the patient, and genomic DNA was extracted as described above. MLPA analysis was performed to detect the copy numbers of exons 7 and 8 of both the *SMN1* and *SMN2* genes using the SALSA MLPA Probemix P060 SMA Carrier kit (P060-B2, MRC-Holland, Amsterdam, the Netherlands) according to the manufacturer’s protocols. This kit contains a total of 21 MLPA probes, including two probes each for SMN1 and SMN2, and 17 reference probes for detecting sequences outside this region. The amplified products were between 154 and 342 nt and separated by capillary electrophoresis. The results were analyzed and compared with positive control DNA samples using Coffalyser. Net software.

### Sanger sequencing of *SMN1*/*SMN2* gene

All exon sequence (exons 1, 2a, 2b, 3, 4, 5, 6, and 7) and exon-intron boundaries of the *SMN1*(NM_000344) or *SMN2*(NM_017411) gene from the patient and her parents were amplified into 9 fragments by using specific primers that were previously reported [59]. The target fragments were amplified using Phanta^®^ Max Super-Fidelity DNA polymerase (Vazyme, Nanjing, China). The amplified products were further purified, and subsequent Sanger sequencing was performed on an ABI Prism 377 DNA Sequencer (Applied Biosystems, Foster City, CA, USA).

### Bioinformatics analysis

In this study, we used the online RNA Splicer tool (https://rddc.tsinghua-gd.org/en/ai/rna-splicer) to predict the splicing patterns of potential splicing variants and evaluated the pathogenicity of novel splicing variants by SpliceAI software.

### RNA extraction, RT-PCR, and mutation identification after cloning

Total RNA from the proband’s peripheral blood samples was extracted using Trizol reagent (Invitrogen, USA) according to the protocol of the manufacturer. In brief, TRIzol reagent was added to the peripheral blood sample to lyse blood cells with a 3:1 volume ratio between the former and the latter. Subsequently, chloroform was added to the mixture from the previous step to separate the RNA into the upper aqueous phase. Finally, RNA was recovered by precipitation using isopropanol. The extracted RNA was determined with a NanoDrop 2000 spectrophotometer (Thermo Scientific, USA). A Takara PrimeScript™ RT reagent Kit (TaKaRa) was used to obtain cDNA from RNA reverse transcription following the manufacturer’s protocol. Given the high homology of *SMN1* and *SMN2*, the PCR products amplified from the cDNA of the *SMN1*/*SMN2* gene in the patient with a forward primer SMN-F (5′-TTGGTACCGAGCTCGGATCCATGGCGATGAGCAGCGGC-3′) and a reverse primer SMN-R (5′-ACGGGCCCTCTAGACTCGAGTTAATTTAAGGAATGTGAGCACCTTC-3′) were cloned into the pMini-CopGFP vector (Beijing Hitrobio Biotechnology Co., Ltd) with a ClonExpress II One Step Cloning Kit (Vazyme, Nanjing, China). This step was performed to confirm whether the identified splicing variant by Sanger sequencing described above has aberrant splicing effects in the mature mRNA of the *SMN1* or *SMN2* gene. The cloned SMN-cDNA plasmid was eventually confirmed by Sanger sequencing.

### Minigene splicing assay

We carried out a minigene splicing assay in vitro to further characterize possible splicing effects resulting from the c.628-3T>G variant in *SMN1*. The minigene region was designed to span exon 4, intron 4, exon 5, intron 5, and exon 6 of the *SMN1* gene (Fig. 3B) amplified from the normal human gDNA with a forward primer (5′-TTGGTACCGAGCTCGGATCCAATGAAAATGAAAGCCAAGTTTCA-3′) and a reverse primer (5′-ACGGGCCCTCTAGACTCGAGCATATAATAGCCAGTATGATAGCCACTC-3′). With a ClonExpress II One Step Cloning Kit (Vazyme, Nanjing, China), the amplified products were cloned into the pMini-CopGFP vector (Beijing Hitrobio Biotechnology Co., Ltd). The cloned wild-type recombinant SMN-plasmid was validated by Sanger sequencing. The mutant plasmids with the c.628-3T>G variant were constructed by recombination of the mutant fragments, which were obtained using the mutagenesis primers *SMN1*-MT-F (5′-ATTCCTTAgAGCCAGGTCTAAAATTCAATGGC-3′) and *SMN1*-MT-R (5′-ACCTGGCTcTAAGGAATATTTCAAAGGAAAATTAACTTA-3′). The successful construction of mutant plasmids was confirmed by Sanger sequencing. Both the wild-type and mutant plasmids were transiently transfected into HEK293T cells with Lipofectamine 2000 (Invitrogen, USA) according to the manufacturer’s protocol. After transfection, cells were cultured for 48 h, and RNA was extracted by TRIzol reagent (Invitrogen, USA). RT-PCR was performed with a forward primer (5′-GGCTAACTAGAGAACCCACTGCTTA-3′) and a reverse primer (5′-ACGGGCCCTCTAGACTCGAGCATATAATAGCCAGTATGATAGCCACTC-3′). Agarose gel electrophoresis was conducted to analyze the amplified PCR fragments, and splicing isoforms were identified by Sanger sequencing.

### Treatment

The most suitable individualized treatment for SMA and best initial approach remain unclear. Considering the treatment cost and goals of avoiding pain caused by injections and starting treatment as early as possible, risdiplam was used for the treatment of this patient after obtaining a definitive genetic diagnosis.

## Data Availability

The identified new variant is submitted to GenBank database, accession numbers # 2754057. Other data supporting the findings of this study are available from the corresponding author on reasonable request.
